# Genotype-phenotype analysis of recombinant chromosome 4 syndrome: an array-CGH study and literature review

**DOI:** 10.1186/1755-8166-6-17

**Published:** 2013-05-02

**Authors:** Morteza Hemmat, Omid Hemmat, Arturo Anguiano, Fatih Z Boyar, Mohammed El Naggar, Jia-Chi Wang, Borris T Wang, Trilochan Sahoo, Renius Owen, Mary Haddadin

**Affiliations:** 1 Cytogenetics Department, Quest Diagnostics Nichols Institute, San Juan Capistrano, CA, USA; 2 Ostrow School of Dentistry, University of Southern California, Los Angeles, CA, USA; 3 Cytogenetics Department, Quest Diagnostics Nichols Institute, 33608 Ortega Highway, San Juan Capistrano, CA, 92690, USA

**Keywords:** Recombinant chromosome 4, Pericentric inversion, Array-CGH, Genotype-phenotype analysis

## Abstract

**Background:**

Recombinant chromosome 4, a rare constitutional rearrangement arising from pericentric inversion, comprises a duplicated segment of 4p13~p15→4pter and a deleted segment of 4q35→4qter. To date, 10 cases of recombinant chromosome 4 have been reported.

**Result:**

We describe the second case in which array-CGH was used to characterize recombinant chromosome 4 syndrome. The patient was a one-year old boy with consistent clinical features. Conventional cytogenetics and FISH documented a recombinant chromosome 4, derived from a paternal pericentric inversion, leading to partial trisomy 4p and partial monosomy of 4q. Array-CGH, performed to further characterize the rearranged chromosome 4 and delineate the breakpoints, documented a small (4.36 Mb) 4q35.1 terminal deletion and a large (23.81 Mb) 4p15.1 terminal duplication. Genotype-phenotype analysis of 10 previously reported cases and the present case indicated relatively consistent clinical features and breakpoints. This consistency was more evident in our case and another characterized by array-CGH, where both showed the common breakpoints of p15.1 and q35.1. A genotype-phenotype correlation study between rec(4), dup(4p), and del(4q) syndromes revealed that urogenital and cardiac defects are probably due to the deletion of 4q whereas the other clinical features are likely due to 4p duplication.

**Conclusion:**

Our findings support that the clinical features of patients with rec(4) are relatively consistent and specific to the regions of duplication or deletion. Recombinant chromosome 4 syndrome thus appears to be a discrete entity that can be suspected on the basis of clinical features or specific deleted and duplicated chromosomal regions.

## Background

Pericentric inversions are observed with varying frequency in all human chromosomes. Breakpoint regions of chromosomal inversions often contain high densities of repetitive DNA sequences, such as *Alu and L1* elements, leading to speculation that they could mediate chromosomal rearrangements and serve as hot spots for non-allelic homologous recombination (NAHR) [1].

During meiosis in carriers, a chromosome containing a large inverted segment and its normal homolog are predicted to form a homosynaptic inversion loop, which leads to optimal pairing of the matching segment [2]. The number of chiasmata in the inverted segment is thought to directly correlate the size of the inverted segment [3-5]. Any odd number of crossovers within the inversion loop leads to the production of two alternate recombinant chromosomes: in one chromosome the distal part of the short arm is duplicated and the distal part of the long arm is deleted; the opposite occurs in the other chromosome. Consequently, two alternative recombinants are theoretically possible among the offspring and generally only one is compatible with life, since, large deletions seem to have a more deleterious effect than large duplications [1,3,6,7].

The chromosome 4 inversion involving sub-band p14~p15 and q35 results in two types of recombinant chromosome 4. Approximately 80% of the viable recombinants are partial 4p duplications and 4q deletions [8]. To date, 10 such cases of recombinant chromosome 4 have been reported [9-18]. We report on a one-year old boy carrying a recombinant chromosome 4 with partial duplication of 4p and partial deletion of 4q, resulting from paternal pericentric inversion of chromosome 4 with breakpoints at 4p15.1 and 4q35.1. The breakpoints and the size of duplicated and deleted segments were studied using conventional chromosome analysis, FISH, and array-CGH. A genotypic-phenotypic correlation analysis was performed between the present case and previously reported cases of rec(4) syndromes, and also between the rec(4), dup(4p), and del(4q) syndromes, to further define the relationship of specific chromosomal rearrangements with clinical features.

### Clinical description

The patient is a one year-old male who presented clinically with developmental delay, dysmorphic features including microcephaly, broad nose with anteverted nares, thin upper lips, abnormal ears, short neck, broad chest, and cardiac and genital anomalies. Both parents were apparently normal; however the father was diagnosed with a pericentric inversion of chromosome 4 by prenatal chromosome analysis. Prenatal testing in the paternal grandmother was medically requested following the earlier death of her daughter due to congenital abnormalities. Grandmaternal chromosome analysis confirmed that the father’s inverted chromosome 4 was inherited from his mother (the grandmother of the index case).

## Results

**Figure 1:**
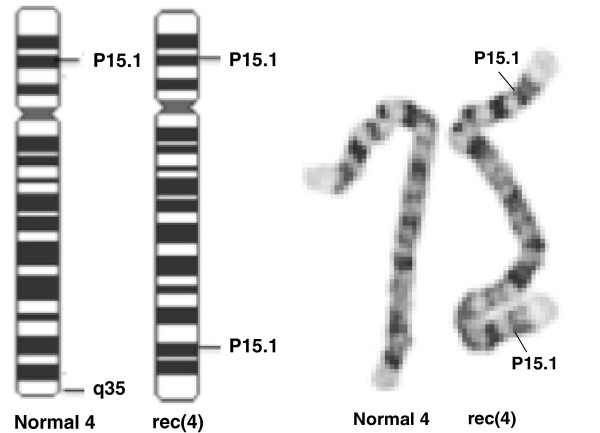
Normal and recombinant chromosome 4 of proband metaphase and their ideograms.

Chromosome analysis of cultured lymphocytes by G-banding revealed 46 chromosomes in all cells, with an abnormal chromosome 4 containing a deletion of 4q35.1-qter and a duplication of 4p15.1-pter. The normal and the recombinant chromosome 4 and their ideograms are shown in Figure 1.

**Figure 2:**
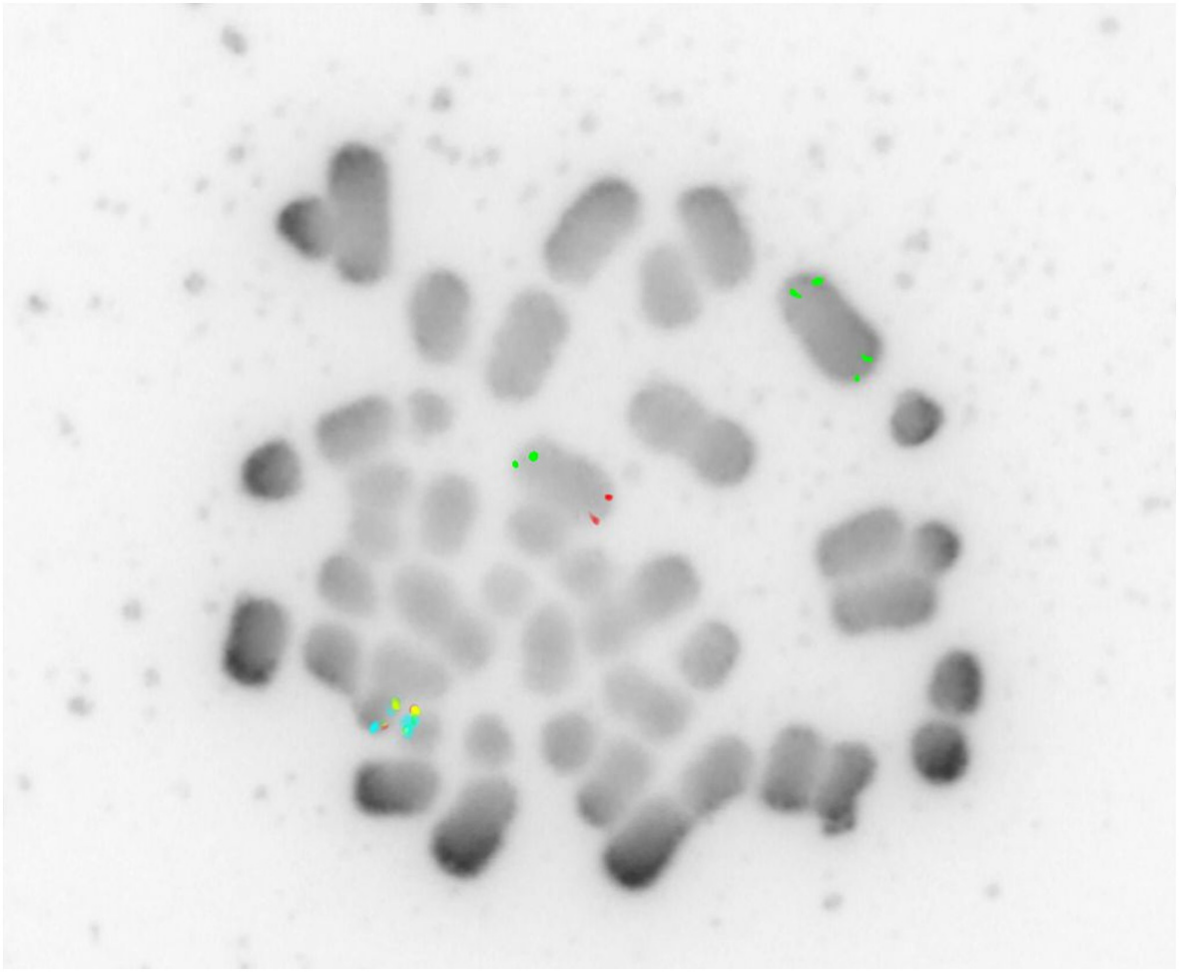
Inverted DAPI image of proband metaphase using Subtel 4p probe (green) and Subtel 4q (red).

Duplication of 4p and deletion of 4q in the recombinant chromosome were also confirmed by using probes specific for sub-telomeric 4p and 4q (Figure 2). Only the normal chromosome 4 showed signals from both 4p and 4q sub-telomeric probes. The recombinant chromosome 4 showed double and symmetrical signals of the 4p sub-telomeric probe but no signal for 4q, since the 4q subtle region was deleted and 4p was duplicated.

**Figure 3:**
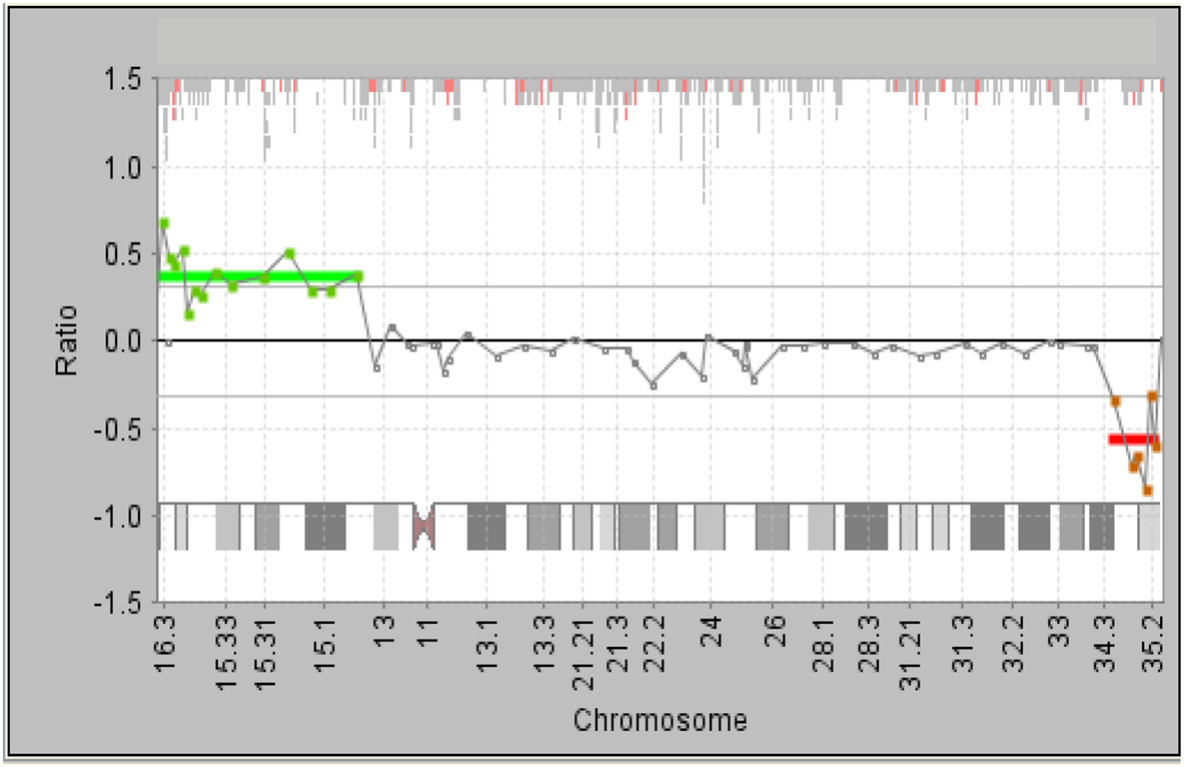
Expanded chromosome 4 aCGH showing 1) a duplicated segment of 4p15.1→4pter with gain of at least 23.81 Mb; and 2) a deleted segment of 4q35.1→4qter with loss of at least 4.36 Mb of distal 4q.

Array-CGH analysis confirmed the partial duplication of 4p15.1-pter and partial deletion of 4q35-qter. The duplicated segment spanned 23.81 Mb, from Bac prob CTD-218L8 to RP11-405D14. The deleted segment spanned 4.36 Mb, from CTB-56N23 to RP11-194A21 (Figure 3). The karyotype of the proband was thus interpreted as 46,XY,rec(4)dup(4p)inv(4)(p15.1q35.1).arr 4p16.3p15.1(CTD-218L8→RP11-405D14)x3,4q35.1q35.2 (CTB-56N23→RP11-194A21)x1 pat.

## Discussion

Recombinant chromosome 4 is a rare chromosomal anomaly. Including our patient, only 11 cases have been described to date [7,10-16,19]. Surprisingly, all cases have the same or very close breakpoints and all inherited the recombinant chromosome 4 from a parent who carried a pericentric inversion of chromosome 4.

A comparison of the breakpoints between patients with recombinant 4 syndrome (Table 1) indicated that all cases had either the same or very close breakpoints, within sub-bands p13~p15 and q35. This consistency was most evident between our patient and another case in which array-CGH was used to more precisely characterize the breakpoints: both showed breakpoints at p15.1 and q35.1. There is increasing evidence for the involvement of repetitive DNA sequences as facilitators of some recurrent chromosomal rearrangements. We suggest that the similarity of breakpoints in all reported rec(4) patients might be due to the presence of these repetitive DNA sequences, which facilitate recurrent pericentric inversions at these chromosomal regions.

**Table 1:** Comparison of the clinical features of the present case and ten other reported cases of recombinant chromosome 4

**Patients**	**Sex**	**Parental inversion**	**Growth retardation**	**Micro-cephaly**	**Pointed chin**	**Broad nose**	**Thin upper lips**	**Abnormal ears**	**Short neck**	**Broad chest**	**Cardio-pathy**	**Genital anomaly**
**1974 (10)**	F	p13q35	+	+	+	+	+	+	+	+	+	-
**1974 (11)**	M	p14q35	+	+	+	+	+	+	+	+	+	+
**1992 (12)**	F	p14q35.2	nr	+	+	+	+	+	+	+	nr	nr
**1993 (13)**	F	p15.32q35	nr	nr	nr	nr	nr	+	nr	nr	nr	nr
**2000 (14)**	F	p16q35.1	+	+	+	+	+	+	+	nr	nr	-
**2002 (15)**	M	p14q35	+	+	+	+	+	+	+	+	+	+
**2002 (7)**	F	p15q35	+	+	+	+	+	+	+	+	+	-
**2007 (16)**	F	p14q35	+	+	+	+	+	+	+	+	-	-
**2007 (16)**	F	p14q35	-	+	+	+	+	+	+	+	-	-
**2009 (21)**	M	p15.1q35	+	-	+	+	+	+	+	+	+	+
**our case**	M	p15.1q35	+	+	+	+	+	+	+	+	+	+

Patients are indicated by reported year and related reference.

F, female; M, male; +, present; -, absent; nr, not reported.

The clinical phenotype of rec(4) has been a subject of debate. In a review of clinical features in patients with rec(4) dup 4p, Garcia-Heras et al. argued that rec(4) dup 4p is not characterized by a clinically recognizable phenotype [7]. Possible reasons include variations in the sizes of the 4q deletion, differences in the breakpoints, and variable expression of the trisomic 4p [7]. In contrast, Bataglia et al. suggested that rec (4) dup 4p appears to be a discrete entity with relatively consistent features [15]. To clarify this argument, we compared the clinical features of all 11 patients with rec(4) reported to date (Table 1). Most of these features, such as growth retardation, microcephaly, abnormal ears, pointed chin, broad chest, short neck, thin upper lips, and broad nose with anteverted nares, were found in all cases. Genital anomalies were reported in all males, and cardiac defects were reported in 5 of 11 patients.

To further evaluate associations between specific chromosome 4 rearrangements and clinical features, we also conducted a genotype-phenotype correlation study of previously reported cases of rec(4), dup(4p), and del(4q) syndromes (Table 2) . Urogenital abnormalities and cardiac defects were common to rec(4) and del(4q) syndrome, indicating their association with the 4q35 deleted region. The consistency of the other clinical features in both rec(4) syndrome cases and dup(4p) cases indicates their association with the 4p15.1 duplicated region. In support of our findings, previous reports [20,21] indicated that cardiac defects are rarely observed in dup 4p patients whose duplication involves the same region affected in rec(4) patients. In addition, Maurin and colleagues [19] found the ArgBP2 and PDLIM3 genes in the 4q35.1 deleted region to be involved in cardiac muscle development. Similarly, urogenital abnormalities, which have rarely been reported in dup(4p) cases, have been consistently reported in 4q deletion syndrome and male rec(4) patients [11,15,19,22]. Therefore, we propose that the genes involved in male genital anomalies are located on 4q.

**Table 2:** **Comparison of the common clinical features of the rec(4)**[7,10-16,19]**, dup(4p)**[20,21]**and del(4q)**[22]**syndromes**

	**rec(4) syndrome**	**dup(4p) syndrome**	**del(4q) syndrome**
**Growth retardation**	+	+	-
**Microcephaly**	+	+	-
**Abnormal ears**	+	+	-
**Pointed Chin**	+	+	-
**Broad chest**	+	+	-
**Short neck**	+	+	-
**Thin upper lip**	+	+	-
**Broad nose**	+	+	-
**Cardiopathy**	+	-	-
**Urogenital abnormality**	+	-	+

+, present; -, absent; nr, not reported.

## Conclusion

In conclusion, our findings support that the clinical features of patients with rec(4) are relatively consistent and specific to the regions of duplication or deletion. Recombinant chromosome 4 syndrome thus appears to be a discrete entity that can be suspected on the basis of clinical features or specific deleted and duplicated chromosomal regions.

## Methods

Peripheral blood samples from a one year-old boy was referred to our laboratory for cytogenetic analysis. Metaphase chromosome preparations were obtained from the patient according to standard procedures. Chromosomes were analyzed with G-banding at the resolution level of 550 bands. To confirm the duplicated and deleted regions, fluorescent in situ hybridization (FISH) analysis was performed with the Tel4pter and Tel4qter probes (Telvysion; Vysis/Abbott, Inc., Downers Grove, IL).

To define the break points and extent of the duplicated and deleted segments, we performed array-CGH using a 1-Mb BAC array with 3222 clones, spaced no more than 1 Mb apart (Quest Diagnostics), scanned on GenePix 4000B microarray scanner (Axon Instruments), and analyzed with Clarisure software. Labeling and hybridization were performed using standard procedures.

### Ethical approval and consent

These studies were performed on anonymized samples received in the clinical laboratory and thus were exempted from the requirement for consent by an opinion for the Western Institutional review Board.

## Abbreviations

rec(4): Recombinant chromosome 4; dup(4p): Duplicated short arm of chromosome 4; del(4q): Deleted long arm of chromosome 4; CGH: Comparative genomic hybridization; Mb: Mega base.

## Competing interest

The authors declare that they have no competing interest.

## Authors’ contribution

MH and OH First co-authors; performed analysis, interpretation of the results, drafting and finalizing the manuscript. TS and MEN participated in writing the case description and revision of the manuscript. BTW, JCW, AA, FZB and RO participated in the revision of the manuscript. All authors read and approved the final manuscript.
